# Management practice and treatment outcomes of adult patients with Lupus Nephritis at the Renal Clinic of St. Paul’s Hospital Millennium Medical College, Addis Ababa, Ethiopia

**DOI:** 10.1186/s12882-022-02846-z

**Published:** 2022-06-17

**Authors:** Gebre-Mariam Tsegay Hailu, Shemsu Umer Hussen, Seifemichael Getachew, Alemseged Beyene Berha

**Affiliations:** 1 Department of Pharmacology and Clinical Pharmacy, School of Pharmacy, College of Health Sciences, Addis Ababa University, Addis Ababa, Ethiopia; 2 Department of Internal Medicine, St. Paul’s Hospital Millennium Medical College, Addis Ababa, Ethiopia

**Keywords:** Lupus nephritis, Treatment outcome, Management practice, St. Paul’s hospital millennium medical college

## Abstract

**Background:**

Lupus nephritis (LN) is the most common severe complication of systemic lupus erythematosus (SLE) which results in high morbidity and mortality. Up to 60% of adult patients with SLE develop the renal disease with different severity. Even with potent anti-inflammatory and immunosuppressive therapies, many LN patients still progress to chronic kidney disease or end-stage renal disease. Thus, this study aimed to assess the management practice, treatment outcomes and to identify the associated factors of poor renal outcome in adult LN patients at the renal clinic of St. Paul’s Hospital Millennium Medical College, Addis Ababa, Ethiopia.

**Methods:**

A retrospective cross-sectional study design was used to collect the data using an abstraction tool from patients’ records. The Kidney Disease Improving Global Outcomes (KDIGO) criteria were used to diagnose LN among SLE patients. Logistic regression was used to determine crude and adjusted odds ratio and a *p*-value of < 0.05 was considered statistically significant. Ethical approval was obtained from the ethical review committee of the School of Pharmacy, Addis Ababa University and institutional review board of St. Paul’s Hospital Millennium Medical College.

**Results:**

Out of 168 study participants enrolled from September 1, 2016 to October 30, 2020, a total of 114 adult LN patients were included for final analysis. The mean (± SD) age of the LN patients at onset was 29.10 ± 9.67 years and 99 (86.8%) of all the patients were females. More than three-fourths (78.9%) of the LN patients had a good prognosis. However, 24 (21.1%) of the patients who didn’t achieve complete or partial remission had a poor prognosis. A kidney biopsy was done for 71 patients at initial presentation with class IV and III as the commonest class. The commonly prescribed immunosuppressive medications were cyclophosphamide as induction therapy in 67 (58.7%) and mycophenolate mofetil (MMF) as maintenance therapy in 76 (66.7%). Gastrointestinal intolerances like abdominal pain, nausea, or diarrhea from MMF were the most common 27(31.2%) treatment-related adverse events reported. Acute kidney injury (AKI) at onset (AOR = 4.83, *P* = 0.026), high serum creatinine (SCr) at six months (AOR = 0.12, *P* = 0.003), no response at six months to attain complete remission (AOR = 0.05, *P* = 0.041) and presence of flare (AOR = 0.04, *P* = 0.004) were predictors poor treatment outcomes.

**Conclusion:**

Despite good response with the present immunosuppressive regimens, relapse, treatment-related complications and adverse events are major problems that require close monitoring**.** The results and identified gaps of this study are used as an input to improve the management practice of LN in the study setting. Overall, this study is comparable with other findings and strengthen the present available literatures.

**Supplementary Information:**

The online version contains supplementary material available at 10.1186/s12882-022-02846-z.

## Background

Systemic lupus erythematosus (SLE) is a severe autoimmune disorder characterized by the involvement of multiple organs. It is an autoimmune disorder in which the immune system attacks its own tissues, causing widespread inflammation and tissue damage within the affected organs. It affects the joints, skin, brain, lungs, kidneys, and blood vessels. The degree or severity of the complications mainly depends on the area affected ranging from skin to various internal organs with a variety of symptoms. Lupus nephritis (LN) denotes a common and severe manifestation of SLE and could be a major factor exerting a negative impact on long-term renal and patient survival [1–3]. Renal involvement occurs in up to 60% of adult patients with SLE and is a major determinant for morbidity and mortality in these patients. Renal involvement in SLE carries a significant risk of morbidity and mortality, which is related to both disease and treatment-related complications [4, 5]. SLE is a potentially severe autoimmune disorder that shows variations in incidence, prevalence, disease activity and prognosis according to race and ethnicity [6]. LN appears to be more prevalent in certain ethnic groups such as Asians, African Americans and Hispanics [7, 8]. 

As evidence showed that the onset of SLE and lupus flares may trigger by certain environment factors. Among these identifying the environmental risk factors associated with SLE is exposure to ultraviolet radiation, certain medication and hormonal preparations that may result in induce inflammatory response, damage cells and appear clinical symptoms by activating the innate and adaptive immune system [9, 10].

Photosensitivity is well known characteristic in both cutaneous and SLE and has been observed in more than 90% of lupus erythematosus patients following ultraviolet radiation. But, sun-induced organ involvement is rarely reported in lupus erythematosus. It’s believed that sunburn-induced keratinocyte necrosis/apoptosis exposed intracellular antigens as trigger for the generation of autoantibodies that finally mediated immune-complex nephritis [11, 12].

In Africa, an increase in the incidence and prevalence of SLE has been observed currently. Few studies indicate that this high frequency in the African population may be related to the high sun-exposure [13, 14].

Immune complex-mediated LN is the most common cause of kidney disease in SLE. Due to an accumulation of autoantibody-containing immune complexes, the kidney becomes modestly or severely inflamed. Thrombotic microangiopathy, lupus podocytopathy, antiphospholipid antibody-induced vascular lesions and tubule interstitial nephritis are other mechanisms that lead to kidney damage. Renal biopsy is the ‘gold standard’ for diagnosis and classification of LN [15, 16]. The clinical course of LN is heterogeneous and varies from mild subclinical disease to an aggressive course that may rapidly progress to end-stage renal disease (ESRD). The nature and severity of the clinical features of LN do not always predict the underlying histological severity. LN is the most common secondary glomerulonephritis leading to ESRD reported in many countries worldwide [17]. The prognosis of LN could be affected by several demographic, clinical, laboratory, and histological variables at disease presentation, as well as the therapeutic modalities used [18].

The overall goal of LN treatment is prevention of ESRD. To prevent ESRD, short-term treatment strategies have focused on complete or partial reversal of the clinical signs of kidney injury [19]. For this purpose, patients should be treated with an induction therapy with cyclophosphamide (CYC), in combination with corticosteroids, has been used effectively to induce remission in LN, but it has considerable adverse effects. Thus, there has been growing interest in the use of mycophenolate mofetil (MMF) as induction therapy, maintenance therapy, or both for patients with LN. Moreover, other immunosuppressive drugs such as tacrolimus or azathioprine (AZA) are now widely used for maintenance treatment of LN [20–22]. The use of immunosuppressive drugs, such as MMF, CYC, AZA, improves LN outcomes. These drugs are frequently used with corticosteroids. Because of their effectiveness in LN, immunosuppressive drugs reduce the cumulative corticosteroids dose and associated side effects. Immunosuppressive drugs differ from each other with respect to safety during pregnancy, administration route, frequency of dosing, and cost [17, 23].

Even if there has been significant improvement in patients and renal survival over the past years, the current immunosuppressive regimens still achieve suboptimal results, with unacceptable high rates of progression to ESRD, disease relapse rate and treatment related complications. Long-term use of high dose immunosuppressive agents has also resulted in significant toxicity [15, 24]. Since immunosuppressive drugs are related to significant adverse events, special care should be tended to prevent infection like pneumocystis jirovecci pneumonia, and to prevent steroid-induced osteoporosis, gastrointestinal bleeding, dyslipidemia and hypertension [25–28].

Moreover, there is a major difference in prevalence, disease severity, treatment response and clinical outcomes between LN patients from different racial and ethnic backgrounds [2]. The severity of disease has been also described to be increased in Africans and therefore the treatment and outcome of African patients with LN has not been rigorously assessed [29].

Treatment of SLE or LN patients in Africa is also restricted by the availability and cost of immunosuppressive drugs, and by the shortage of facilities for laboratory monitoring of patients like some serological tests and kidney biopsy. Thus, in resource limited countries, clinicians treating LN patients have limited choices of therapy and this may result in poor treatment outcomes [30].

In addition to the high cost and limited availability of drugs, adherence to treatment is a problem among SLE or LN patients worldwide. Moreover, low levels of formal education and long distances to travel for specialist care, contribute to low follow-up rates and poor treatment outcomes [31].

Reducing morbidity and mortality associated with LN patients will require more rapid and complete control of inflammatory kidney injury and minimization of LN flares. To improve the outcome of treatment in such patients, earlier diagnosis and treatment, identifying effective/safe drugs and improving patient compliance are important strategies. Thus, the present study aimed to assess the pattern of response to treatment in adult LN patients and to identify the associated factors for poor renal outcome at the renal clinic of St. Paul’s Hospital Millennium Medical College, Addis Ababa, Ethiopia. The results and identified gaps of this study will be used as an input for clinicians to improve the management of LN in Ethiopian health care settings.

## Methods

### Study setting

The study was conducted at the renal clinic of St. Paul’s Hospital Millennium Medical College (SPHMMC) on patients diagnosed with LN. SPHMMC is located in Gulele Sub-city, Addis Ababa, Ethiopia, was inaugurated in 1968. SPHMMC provides healthcare and trains students in different biomedical and clinical departments. While the inpatient capacity is more than 700 beds, provides a service for an average of 1200 emergency and outpatient clients daily. The renal clinic also provides services on average 15 LN patients of follow up per month. Medical coverage for LN patients is provided by government (health insurance) or self-funded.

### Study design and period

A retrospective hospital based cross sectional study design was used to collect the data from October 26, 2020 to January 15, 2021. Data was collected by reviewing the medical records of lupus nephritis patients who attended the renal unit of SPHMMC from September 1, 2016 to October 30, 2020.

### Source and study population

The source population was all LN patients who visited the renal clinic of St. Paul’s Hospital Millennium Medical College. All LN patients who had a follow up in the renal clinic of SPHMMC during the study period and those who fulfilled the inclusion criteria were recruited.

### Sampling and sample size determination

All adult LN patients who attended in the renal clinic of SPHMMC during the study period were included as the study participants. Convenience sampling method was used to collect the necessary data that fulfills the inclusion criteria.

### Inclusion and exclusion criteria

All lupus nephritis patients (SLE patients with renal involvement) that fulfills the Kidney Disease Improving Global Outcomes (KDIGO) criteria, age ≥ 18 years and patients who had at least six months of follow up at the clinic were the inclusion criteria.

Any glomerulonephritis (GN) not associated with SLE and patients who had incomplete medical records or lost to follow up were the exclusion criteria.

### Data collection procedures and instrument

The data abstraction tool was developed after reviewing similar published articles previously. A data abstraction format/tool was used to collect the necessary information from patient charts records. Health management information system (HMIS) patient registration book was used for accessing the card numbers.

Two clinical pharmacists were employed as data collectors. Prior to data collection, a one-day training was given to the data collectors about the aim of the study and detailed review of the data collection tool. The training was followed pre-testing of the tool by 5% of the sampling population. The investigators and the data collectors were then discussing lessons learnt from the pre-test to modify and include the necessary information in the tool for further clarify some of the issues during the data collection process. Throughout the data collection process, the research team did close supervision. The collected data was checked on regular basis for completeness and consistency.

### Data analysis and interpretation

First, the data were checked for completeness and consistency. The data were cleaned, then entered to Statistical Package for Social Science (SPSS) window version 25 for analysis. Descriptive statistics included mean and standard deviation for continuous variables and frequency and percentage for categorical data was used to summarize socio-demographic and clinical characteristics of the study participants. Logistic regression analysis was performed to identify the independent predictors of treatment outcome of LN patients. After checking the absence of collinearity among variables, variables in univariate analysis with *p*-value ≤ 0.25 were further analyzed in multivariate logistic regression to control the effect of confounders. Odds ratio (OR) was used to measure association of dependent and independent variables where 95% confidence interval (CI) and *P* < 0.05 value was utilized to determine statistical significance.

### Operational definition

#### Lupus nephritis

An inflammation which affects the nephrons within the kidneys as a result of the complication of SLE. The patient should fulfill the diagnosis of KDIGO criteria. These includes; proteinuria of 0.5 g/d and above, serum creatinine of 1.5 mg/dl and above or evidence of decreased or decreasing eGFR, active urinary sediment (≥ 5% RBC/HPF, ≥ 5% WBC/HPF cellular casts) and biopsy-proven LN [25, 27].

#### Complete renal remission

The KDIGO guideline defines complete remission as a reduction in proteinuria to < 0.5 g/g measured as the urine protein to creatinine ratio (UPCR) from 24-h urine collection and stabilization or improvement in kidney function (± 10 – 15% of baseline) within 6 – 12 months of starting therapy, but could take more than 12 months [27].

#### Partial renal remission

According to the KDIGO guideline partial remission is defined by a reduction in proteinuria by at least 50% and to < 3 g/g measured as the UPCR from 24-h urine collection and stabilization or improvement in kidney function (± 10 – 15% of baseline) within 6 – 12 months of starting therapy [27].

#### No response/non responders

Failure to achieve a partial or complete response within 6 – 12 months of starting therapy [27].

#### Baseline serum creatinine

The initial value of serum creatinine recorded.

#### Good prognosis

Patients that achieve complete or partial remission during the study period are used as favorable clinical outcomes.

#### Poor prognosis

Patients that have no response, progression to ESRD or death at the end of the study period are used as unfavorable clinical outcomes.

#### Hypertension

Blood pressure above or equal to 140/90 mmHg or using of antihypertensive medications on regular follow-up [32, 33].

#### Leucopenia

WBC count of < 4000/mm^3^ in the absence of other causes (at least once ruled out other causes).

#### Thrombocytopenia

Platelet count of < 100,000/mm^3^ at least once ruled out other causes.

#### Flare/relapse

Defined as increase in active urinary sediments, proteinuria, and serum creatinine in patients who were previously in CR or PR.

#### Acute kidney injury (AKI)

Defined as an abrupt decline in renal function, clinically manifesting during the LN diagnosis or after that based on increase in serum creatinine of ≥ 0.3 mg/dl or ≥ 50% within 48 h or urine output of < 0.5 ml/kg/hours for > 6 h over the course of hours to weeks.

#### Adverse drug events

Harmful and unintended consequences of medication use that includes adverse drug reactions or medication errors [34].

## Results

### Socio-demographic, clinical and laboratory characteristics of patients

From 168 study participants enrolled in this study, a total of 114 LN patients were included for final analysis and 54 were excluded because of incompleteness of data as well as lost to follow up. The mean (± SD) age at onset of LN was 29.10 ± 9.67 years and 99(86.8%) of all the patients were females with a female-to-male ratio of 6.6:1. More than half (56.1%) of the patients were lived in rural area. Out of the total 3(2.6%) of patients were smokers but the remaining was not known their status. Other socio-demographic characteristics like marital status, educational status and monthly income couldn’t found from the patient’s chart. The baseline socio-demographic characteristics are shown below (Table 1).

**Table 1:** Socio-demographic characteristics of LN patients at the renal clinic of St. Paul’s Hospital Millennium Medical College, Addis Ababa, Ethiopia from Sep, 2016 to Oct, 2020 (*n* = 114)

Variable		Frequency (%)	Mean ± SD
Gender	Male	15(13.2)	
	Female	99(86.8)	
Age (years)	18 – 29	64(56.1)	
	30 – 49	46(40.4)	
	50 – 64	4(3.5)	
Residence	Rural	64(56.1)	
	Urban	50(43.9)	
Mean follow up period (months)			27.93 ± 17.15

At onset of LN patients, 97(85.1%) had body swelling (edema) and 53(46.5%) were hypertensive. Nephrotic syndrome or nephrotic range proteinuria was found in 76(66.7%) of LN patients at the initial presentation. Hematuria was found in 86(75.4%) of LN patients at the initial presentation. Acute kidney injury was also found in 48(42.1%) of LN patients as a complication at the initial presentation. Leucopenia 29(25.4%) and thrombocytopenia 19(16.7%) was found the common hematologic manifestations in LN patients. Antiphospholipid syndrome was diagnosed in 5(4.4%) of LN patients at the initial presentation.

Central nervous system manifestations as lupus cerebrities, skin manifestations as discoid lupus erythematous, pleural/pericardial effusion and rheumatoid arthritis were the common extra renal manifestations in these LN patients. Baseline clinical and laboratory parameters are shown in Table 2.

**Table 2:** Clinical characteristics and co-morbid diseases of LN patients at the renal clinic of St. Paul’s Hospital Millennium Medical College, Addis Ababa, Ethiopia from Sep, 2016 to Oct, 2020 (*n* = 114)

Baseline characteristics	Mean ± SD	Frequency (%)
Age at diagnosis	29.10 ± 9.67	
Edema at onset (%)		97(85.1)
Hypertension at onset (%)		53(46.5)
Nephrotic syndrome (≥ 3.5 g/ 24 h urine protein) (%)		76(66.7)
Antiphospholipid syndrome (%)		5(4.4)
CNS manifestations (lupus cerebrities) (%)		10(8.8)
Skin manifestations (discoid lupus erythematous) (%)		13(11.4)
Rheumatoid arthritis (%)		15(13.2)
Pleural/pericardial effusion (%)		22(19.3)
Thrombocytopenia (%)		19(16.7)
Leucopenia (%)		29(25.4)
Thrombosis/venous (%)		6(5.3)
Anemia (%)		4(3.5)
Hematuria (%)		86(75.4)
AKI (acute tubular necrosis, interstitial nephritis) (%)		48(42.1)
UTI and renal stone (%)		7(6.1)
Infection-related glomerulonephritis (%)		12(10.5)
Tuberculosis (%)		7(6.1)
Other nonspecific symptoms (%)^a^		21(18.4)

*SD* standard deviation, *CNS* central nervous system, *AKI* acute kidney injury, *UTI* = urinary tract infection

^a^Other symptoms (rash, oral ulcers, fever, fatigue)

At the time of diagnosis of LN, mean (± SD) SBP and DBP were 129.52 ± 19.96 and 82.59 ± 14.32 mmHg, respectively. The mean (± SD) baseline serum creatinine was 2.45 ± 2.17 mg/dL and the mean (± SD) baseline 24-h urine protein was 4.47 ± 2.24 g/day. At the initial presentation the mean (± SD) estimated glomerular filtration rate (eGFR) calculated using the Chronic Kidney Disease Epidemiology Collaboration (CKD-EPI) was 58.36 ± 42.31 ml/min. But 34(29.8%) was classified as stage 3 CKD patients and other baseline laboratory test results are shown in Table 3.

**Table 3:** Baseline laboratory results of LN patients at the renal clinic of St. Paul’s Hospital Millennium Medical College, Addis Ababa, Ethiopia from Sep, 2016 to Oct, 2020

Variables	Number of patients (n)	Mean ± SD or %
Hg (mg/dl)	114	11.80 ± 2.94
WBC (× 10^3^/mm^3^)	114	7.40 ± 3.60
PLT (× 10^3^/mm^3^)	114	259.88 ± 134.24
ESR (mm/hour)	44	56.22 ± 36.51
Serum albumin (mg/dl)	53	2.77 ± 0.64
Creatinine (mg/dl)	114	2.45 ± 2.17
CKD-EPI eGFR (ml/min)	114	58.36 ± 42.31
Urea (mg/dl)	114	81.79 ± 52.42
Urine proteins (g/24 h)	114	4.47 ± 2.24
ANA (positive)	85	74.6
Anti-dsDNA (positive)	31	27.2
C3 (low)	44	38.6
C4 (low)	38	33.3
LA (positive)	11	9.6
SBP at onset of LN (mmHg)	114	129.52 ± 19.96
DBP at onset of LN (mmHg)	114	82.59 ± 14.32
Lupus class/kidney biopsy (%)	71	
Class II		3(4.2)
Class III		20(28.2)
Class IV		28(39.4)
Class V		5(7.0)
Class III/V		7(9.9)
Class IV/V		8(11.3)
CKD-EPI eGFR Stages (%)	114	
Stage 1		25(21.9)
Stage 2		19(16.7)
Stage 3		34(29.8)
Stage 4		20(17.5)
Stage 5		16(14.0)
Mean follow up period (months)	114	27.93 ± 17.15

*SD* standard deviation, *Hg* hemoglobin, *WBC* white blood cells, *PLT* platelets, *ESR* erythrocyte sedimentation rate, *CKD-EPI eGFR* chronic kidney disease epidemiology collaboration estimated glomerular filtration rate, *ANA* antinuclear antibody, *Anti-dsDNA* anti-double strand DNA, *C3&C4* complement levels, *LA* lupus anticoagulants, *SBP* systolic blood pressure, *DBP* diastolic blood pressure

Kidney biopsy was done for 71 patients at initial presentation and most of them were classified as class IV 28(39.4%) followed by class III 20(28.2%). Serology tests done at the onset includes; 85(74.6%) were antinuclear antibody (ANA) positive, 31(27.2%) were anti-double-stranded DNA antibody (Anti-dsDNA) positive and 11(9.6%) were lupus anticoagulants (LA) positive. Complement level determinations were also done for C3 (low) and C4 (low) which accounts for 44(38.6%) and 38(33.3%), respectively.

### Treatment regimen and treatment associated adverse events

Different immunosuppressive regimens were used as induction and maintenance phase therapy in confirmed or presumed LN patients. Most proliferative LN were given pulse steroids with 500–1000 mg IV infusion methylprednisolone or oral prednisolone at 2 mg/kg for 3 days and then continued with prednisolone of 1 mg/kg/day. After that prednisolone is tapered at 1 mg/kg within one to three months according to the response criteria for LN or LN disease activity. In stable patients’ prednisolone was tapered to 5 mg daily and continued indefinitely as maintenance. The induction and maintenance regimens were depicted in Table 4.

**Table 4:** Immunosuppressive regimens used for induction and maintenance phase therapy in LN patients at the renal clinic of St. Paul’s Hospital Millennium Medical College, Addis Ababa, Ethiopia from Sep, 2016 to Oct, 2020 (*n* = 114)

Phase (type of therapy)	Frequency (%)
**Induction**	
Pred only	6(5.3%)
Pred + CYC	67(58.7%)
Pred + MMF	34(29.8%)
Pred + TAC	3(2.6%)
Pred + TAC + MMF	2(1.8%)
Pred + RTX	8(7.1%)
**Maintenance**	
Pred + MMF	76(66.7%)
Pred + AZA	14(12.2%)
Pred + CYC	32(28.1%)
Pred + TAC	1(0.9%)
Pred + MMF + TAC	1(0.9%)
Pred + RTX	4(3.5%)

*Pred* prednisolone, *CYC* cyclophosphamide, *MMF* mycophenolate mofetil, *TAC* tacrolimus, *AZA* azathioprine, *RTX* rituximub

CYC was given as induction treatment for 67(58.7%) LN patients followed by 34(29.8%) was taken MMF. For the maintenance therapy 76(66.7%), 32(28.1%) and 14(12.2%) of LN patients took MMF, CYC and AZA, respectively. Rituximab and tacrolimus were given in refractory LN patients. CYC dosing was based on the National Institute of Health (NIH) regimen, which is IV 0.5 – 1 g/m2 monthly for six months. MMF starting dose was 500 to 1000 mg twice daily according to their disease activity. AZA dose was given from 50 to 100 mg daily.

Adverse events reported from the different regimens during the study period according to the treating physicians include gastrointestinal (GI) intolerance (abdominal pain, nausea, diarrhea), peptic ulcer, cushingoid appearance, diabetes mellitus, leucopenia, psychosis, cataract/glaucoma, infection (candidiasis, herpes, urinary tract infection) and pleural effusion. GI intolerance presented as abdominal pain, nausea or diarrhea was the most common 27(31.2%) adverse effect followed by leucopenia 15(17.4%). The GI intolerance manifested as abdominal pain, nausea, and diarrhea was reported as the side effects of MMF. Cushingoid appearance, diabetes mellitus, peptic ulcer, psychosis and cataract/glaucoma was reported due to steroid (prednisolone) use. Leucopenia was reported from cyclophosphamide, MMF and AZA. Ocular disorder also reported from the use of chloroquine. Table 5 below indicates the different types of adverse events reported.

**Table 5:** Adverse events occurred during the treatment of LN patients at the renal clinic of St. Paul’s Hospital Millennium Medical College, Addis Ababa, Ethiopia from Sep, 2016 to Oct, 2020 (*n* = 86)

Adverse events	Frequency (%)
Cushingoid appearance	9(10.5)
Diabetes mellitus	8(9.3)
Peptic ulcer	11(12.8)
Psychosis	7(8.1)
Cataract/glaucoma	4(4.7)
GI intolerance (abdominal pain, nausea, diarrhea)	27(31.2)
Leucopenia	15(17.4)
Infection (candidiasis, herpes, UTI)	4(4.7)
Pleural effusion	1(1.2)

*GI* gastrointestinal, *UTI* urinary tract infection

### Management practice of comorbidities and complications in LN patients

In the management of LN patients’ adjunctive therapies should be considered to minimize risk of complications related from the disease or from the regimens. It was found that 98(86%) of LN patients were taken the available antimalarial therapy chloroquine in our setup. The dose of chloroquine given is 250 mg on daily basis. Kidney protective regimens with either angiotensin enzyme inhibitor (ACEI) or angiotensin receptor blockers (ARBs) were prescribed to 87.7% of LN patients. The most common ACEI/ARBs prescribed in this study setting were enalapril, lisinopril, losartan and irbesartan with tolerated dose (See in Table 6).

**Table 6:** Other medications/adjuvant drugs prescribed for co-morbid disease for LN patients at the renal clinic of St. Paul’s Hospital Millennium Medical College, Addis Ababa, Ethiopia from Sep, 2016 to Oct, 2020 (*n* = 114)

Medications	Frequency (%)
Anti-malarial therapy ^a^	98(86)
Kidney protective regimens ^b^	100(87.7)
Antihypertensive drugs ^c^	70(61.4)
Diuretics ^d^	72(63.2)
Antilipemic agents ^e^	38(33.3)
Anticoagulants ^f^	39(34.2)
Prophylaxis for pneumocystis jiroverci pneumonia^g^	77(67.5)
PPIs/antacids ^h^	66(57.9)
Antidiabetics ^i^	10(8.8)
Antianemic agents ^j^	22(19.3)
Dermatologic agents ^k^	27(23.7)
Anticonvulsants/antidepressants ^l^	3(2.6)

^a^ chloroquine

^b^ ACEIs (enalapril, lisinopril), ARBs (losartan, irbesartan)

^c^ calcium channel blockers (amilodipine, nifedipine, verapamil), beta blockers (metoprolol, carvedilol, labetalol, atenolol), hydralazine

^d^ furosemide, hydrochlorothiazide, spironolactone

^e^ atorvastatin, simvastatin

^f^ warfarin, UFH, enoxaparin, aspirin, tranexamic acid

^g^ cotrimoxazole

^h^ omeprazole, pantoprazole, esmoprazol, ranitidine

^i^ insulin, metformin

^j^ ferrous sulphate, ferrous gluconate, epoitin alpha, cyanocobalamine

^k^ fluconazole, terbinafin, miconazole, sunscreens and emollients

^l^ sertraline, phenytoin, diazepam

*ACEIs* angiotensin converting enzyme inhibitors, *ARBs* angiotensin receptor blockers, *UFH* unfractionated heparin, *PPIs* proton pump inhibitors

Antihypertensive agents in LN patients were used to achieve blood pressure goal and to control proteinuria. The most common antihypertensive drugs prescribed for LN patient includes calcium channel blockers, beta blockers, diuretics and hydralazine. The most prescribed antilipemic agents were atorvastatin and simvastatin. The common anticoagulants prescribed to LN patient include warfarin, unfractionated heparin (UFH), enoxaparin, aspirin and tranexamic acid. Cotrimoxazole was prescribed in 67.5% of LN patients for pneumocystis jiroverci pneumonia prophylaxis.

Chronic use of steroid in both high and low dose is associated with a lot of complications. In our study 57.9% of LN patients were used proton pump inhibitors (PPI) for peptic ulcer prophylaxis. The other common medications used for comorbidities and complications of LN patients are antidiabetic agents, antianemic agents, antirheumatics, dermatologic agents and antituberculosis.

### Treatment outcome

**Fig. 1:**
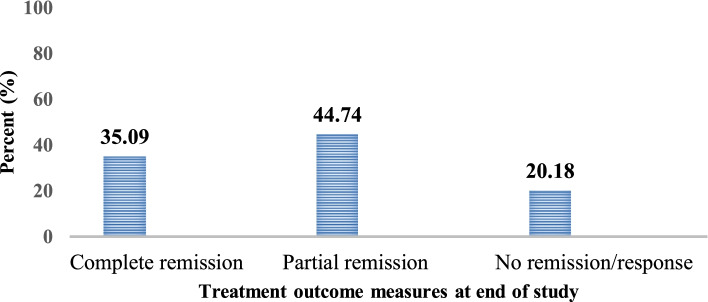
Outcome of the treatment of LN patients at the renal clinic of St. Paul’s Hospital Millennium Medical College, Addis Ababa, Ethiopia from Sep, 2016 to Oct, 2020 (*n* = 114)

**Fig. 2:**
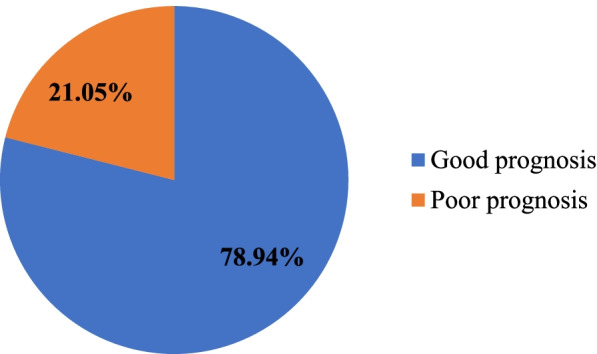
Good and poor prognosis outcomes of LN patients at the renal clinic of St. Paul’s Hospital Millennium Medical College, Addis Ababa, Ethiopia from Sep, 2016 to Oct, 2020 (*n* = 114)

As shown in Fig. 1, 40(35.1%) patients achieved complete remission, 7(6.14%) patients progressed/reached to ESRD and death occurred in 4(3.51%) patients. Moreover, out of the total LN patients, more than three-fourth (78.9%) of them had a good prognosis, that have a complete or partial remission (See in Fig. 2).

Exacerbation or worsening of edema 23(29.9%) and relapse 20(26.0%) were found the common hospitalization events and reason for admission during the study period (See in Table 7).

**Table 7:** Hospitalization events and reason of admissions in LN patients at the renal clinic of St. Paul’s Hospital Millennium Medical College, Addis Ababa, Ethiopia from Sep, 2016 to Oct, 2020 (*n* = 77)

Hospitalization events	Frequency (%)
Due to LN flare / relapse	20(26.0)
Exacerbation / worsening of edema	23(29.9)
Acute kidney injury	11(14.3)
Increased blood pressure	6(7.8)
Infection	11(14.3)
Deep vein thrombosis	2(2.6)
Severe anemia	4(5.2)

### Factors associated with treatment outcome

Univariate analysis showed that gender, hypertension at onset, AKI at onset, baseline SCr value, baseline 24-h urine protein, six-month SCr value, response at six-month, hospitalization events and presence of flare have been revealed p-value which was less than 0.25 (Table 8) and they were incorporated for multivariate binary logistic regressions. According to the multivariate analysis, four variables were significantly associated with the treatment outcomes. Those statistically significant correlations with the treatment outcomes were found in AKI at onset, six-month SCr value, response at six-month and presence of flare.

**Table 8:** Univariate and multivariate logistic regression analysis of factors associated with treatment outcome among patients with lupus nephritis on follow up at St. Paul’s Hospital Millennium Medical College, Addis Ababa, Ethiopia from Sep, 2016 to Oct, 2020 (*n* = 114)

Outcome variables	Treatment outcome		Univariate analysis		Multivariate analysis	
	Good prognosis, n (%)	Poor prognosis, n (%)	COR(95%CI)	*P*-value	AOR(95% CI)	*P*-value
Gender						
Male	14 (12.3)	1 (0.9)	0.236(0.029–1.892)	0.17	0.07(0.003–1.537)	0.091
Female	76 (66.7)	23 (20.2)	1.000		1.000	
Hypertension at onset						
Yes	38(33.3)	15(13.2%)	0.438(0.174–1.107)	0.08	0.58(0.155–2.196)	0.425
No	52(45.6)	9(7.9)	1.000		1.000	
AKI at onset						
Yes	30(26.3)	18(15.8)	6.00(2.158–16.683)	0.001	4.83(1.207–19.286)	0.026^*^
No	60(52.6)	6(5.3)	1.000		1.000	
Baseline SCr. (mg/dl)						
> 1.5 mg/dl	44(38.6)	18(15.8)	0.319(0.116–0.877)	0.027	0.73(0.195–2.760)	0.647
< 1.5 mg/dl	46(40.4)	6(5.3)	1.000		1.000	
Baseline urine proteins (g/24 h)						
> 3.5 g	58(50.9)	20(17.5)	3.036(0.956–9.635)	0.06	1.15(0.258–5.171)	0.851
< 3.5 g	32(28.1)	4(3.5)	1.000		1.000	
Six month SCr value						
> 1.5 mg/dl	12(10.5)	16(14.0)	0.077(0.027–0.218)	0.000	0.12(0.030–0.475)	0.003^*^
< 1.5 mg/dl	78(68.4)	8(7.0)	1.000		1.000	
Response at six month						
CR	23(20.2)	1(0.9)	0.127(0.016–0.991)	0.049	0.05(0.003–0.891)	0.041^*^
No CR	67(58.8)	23(20.2)	1.000		1.000	
Hospitalization events						
Yes	29(25.4)	15(13.2)	3.506(1.373–8.950)	0.009	0.32(0.054–1.846)	0.200
No	61(53.5)	9(7.9)	1.000		1.000	
Presence of flare						
Yes	8(7.0)	12(10.5)	0.098(0.033–0.287)	0.000	0.04(0.005–0.374)	0.004^*^
No	82(71.9)	12(10.5)	1.000		1.000	

*COR* crude odds ratio, *AOR* adjusted odds ratio, *CI* confidence interval, *AKI* acute kidney injury, *SCr* serum creatinine, *CR* complete remission

^*^significant association (*p* < 0.05)

LN patients presented with initial AKI or later as a complication was result in poor prognosis (Adjusted Odds Ratio (AOR) = 4.83; 95% CI: 1.207–19.286, *P* = 0.026)). From the AOR for AKI indicates that patients who had AKI were found to be 4.8 times higher odds of poor prognosis than those without. A significant association was found between poor prognosis of LN patients and the six-month value of SCr (AOR = 0.12; 95% CI: 0.030–0.475, *P* = 0.003). LN patients who had increased six-month value of SCr were found 12% higher risk of poor prognosis than those who had decreased six-month SCr from the baseline. Complete remission at six-month results in a good prognosis at the end of treatment (AOR = 0.05; 95% CI: 0.003–0.891, *P* = 0.041). Any history of relapse or flares during treatment of LN patients results in poor prognosis and for additional immunosuppressive treatments (AOR = 0.04; 95% CI: 0.005–0.374, *P* = 0.004).

## Discussion

In this study, medical records of 114 LN patients were retrospectively evaluated to assess the management practice and treatment outcome of LN. All the patients were evaluated using the KDIGO criteria for the treatment outcome and associated factors. Based on these criteria 78.9% of the LN patients had good prognosis and 21.1% patients were found to have poor prognosis. According to the results of this study females were found dominant in number with a female-to-male ratio of 6.6:1 and the mean (± SD) age at onset of LN was 29.10 ± 9.67 years. The age range in this study was from 18 – 63 years and 56.1% of the patients were found below 30 years. The gender ratio is lower compared to studies done in Morocco, 7.8:1 [35], South Africa, 7.4:1 [36], Southern India, 8:1 [37] and Saudi Arabia, 8.3:1 [38] but higher compared to Egypt, 5.4:1 [39], Tunisia, 5.8:1 [40], Senegal, 4.3:1 [41] and Jordan, 6.2:1 [42]. The age distribution is comparable and slightly lower compared to studies from Morocco [35], Jordan [42], South Africa [43], Kenya [44], Saudi Arabia [38] and Senegal [41]. But the mean age in this study is slightly higher than the results of a study in Tunisia [40] and Southern India [37]. This gender and age distribution difference may be due to variation in study participants, study design, socioeconomic status and health care practice in screening and diagnosing of SLE patients for renal involvement follow up. The reason why LN is more dominant in females during childbearing age is that due to the exposure to high endogenous estrogen level which may increase the risk for the development of the disease. This is also supported by other study [45].

Edema at onset 97(85.1%), nephrotic syndrome 76(66.7%), hypertension 53(46.5%), hematuria 86(75.4%) and AKI 48(42.1%) were found the common initial clinical presentation in LN patients in this study. At the time of diagnosis of LN, mean SBP and DBP were 129.52 ± 19.96 and 82.59 ± 14.32 mmHg, respectively. This finding is similar to the results of a study done in Morocco where nephrotic syndrome (52.6%), hypertension (33.3%) and hematuria (76.3%) were the initial manifestations [35]. Another study from South Africa reported that 54.8 and 31.0% of all patients had edema and hypertension, respectively, at onset of LN [36]. The study done in India reported 33.3% of the patients had hypertension and 34% nephrotic range proteinuria at the initial presentation [37]. Edema, nephrotic syndrome, hypertension and hematuria, the most common initial clinical presentations in LN patients, were found similar with other studies [40–43, 46].

In this study, leucopenia 29(25.4%) and thrombocytopenia 19(16.7%) were found the common hematologic manifestations/disorders in LN patients. A study done by Shivaprasad et al.,in India reported similar findings [37]. But this hematological manifestations is higher than the results of a study from Egypt [47]. The possible reason for this variation may be due to the difference in ethnicity, presence of comorbidities or complications at diagnosis and the type of medication taken.

The mean baselines of SCr, serum albumin and 24-h urine protein were 2.45 ± 2.167 mg/dL, 2.77 ± 0.64 mg/dL and 4.47 ± 2.24 g/24 h, respectively. At the initial presentation, the mean eGFR calculated using the CKD-EPI was 58.36 ± 42.31 ml/min and majority of the patients (29.8%) were classified as stage 3 CKD. These findings are comparable to the results of a study done in India [37]. A study by Okpechi et al., in South Africa reported that the mean baseline eGFR is higher [36] compared to the present study. In addition, a similar study from Senegal, 2020 [48] reported that the baseline eGFR is slightly higher. This discrepancy could be due to the difference in the study participants, the type of medication used and the formula used to calculate eGFR using MDRD and CKD-EPI.

Kidney biopsy was done for 71(62.3%) patients at initial presentation and most of them were classified as class IV 28(39.4%) followed by class III 20(28.2%). This is similar to the findings in India [37], Egypt [47], South Africa [43], Tunisia [40] and Senegal [41] as class IV and class III were the commonest kidney biopsy in LN patients at the initial presentation.

A study in Jordan class IV and V were the most common pathological class of LN but class III are lower than [42] to this study. A study by Niang et al., in Senegal indicates that class IV and V were found the commonest [46]. In the present study class V were lower compared to studies done in South Africa [36], Egypt [39], Senegal [48] and London [49]. This variation could be due to the availability and affordability of kidney biopsy in the study setting is limited.

In this study immunologic tests done at disease onset includes; 85(74.6%) were ANA positive, 31(27.2%) were Anti-dsDNA positive and 11(9.6%) were LA positive. Complement level determinations were also done for C3 (low) and C4 (low) in 44(38.6%) and 38(33.3%) respectively. These serologic tests were lower compared to other studies conducted [35–37, 40, 43, 49]. The possible reason for this variation could be due to the difference on the availability and physicians’ choice of diagnostic tests. In addition, it may be due to variation in study design and study participants.

The treatment regimen used for the different classes of LN for induction and maintenance therapies as well as other adjuvant drugs used were assessed in this study. According to this CYC and MMF were given as induction treatment and maintenance therapy coupled with 67(58.7%) and 34(29.8%) patients, then 76(66.7%) and 32(28.1%) patients, respectively. AZA was given in 14(12.2%) as a maintenance therapy. In addition, rituximab and tacrolimus were given in refractory LN patients. Prednisolone was used in all patients. This is similar to the finding in Texas, 2011 most patients received IV CYC for induction, few use MMF but most patients use MMF as maintenance therapy and few use IV CYC for maintenance therapy [24].

In the present study the use of MMF as induction and maintenance therapy is higher compared to previous studies done in Africa; most of them used CYC as induction treatment [29]. This may indicate good adherence to recent clinical practice guidelines in the study setting. The present study findings in line with study done in South Africa on 87 LN patients [50].

A study from Senegal in 2020 reports that at the induction phase most patients received steroids (with pulse methylprednisolone for 3 days followed by an oral prednisone) for a total of 99 LN patients [48]. This is similar to the present study on the choice of immunosuppressive drugs for the induction and maintenance therapy. On the other hand, study in South Africa for outcome of patients with membranous LN indicates that prednisolone plus CYC used commonly as induction. Also, more patients received prednisone and azathioprine for maintenance therapy. Few patients received prednisolone plus CYC as maintenance and MMF or prednisolone alone as maintenance [36]. This is also supported by a study done in Eastern India for short-term outcomes of LN patients uses CYC and MMF as induction agent [51].

In this study, most LN patients receive chloroquine, ACEI/ARB, anti-platelet agents and lipid lowering drugs as adjuvant therapy as supported by a study in South Africa [36]. This is also in line with the KDIGO 2021 guideline recommends that patients with LN should be treated with hydroxychloroquine or an equivalent antimalarial (chloroquine) unless contraindicated. It also recommends kidney protective therapy using RAAS blockage in LN patients is the key principle of patients to prevent progression to ESRD [27].

GI intolerance presented as abdominal pain, nausea or diarrhea was the most common 27(31.2%) adverse event reported from the use of MMF in this study. Diarrhea was reported as the frequent adverse event of MMF supported by other studies [52, 53]. A study by Lu et al., done in active LN patients reports that 4.2% suffered from gastrointestinal upset as a side effect of MMF which resolved without discontinuation [54]. In addition, in this study, according to the treating physicians’ the following adverse events were reported: peptic ulcer, cushingoid appearance, diabetes mellitus, leucopenia, psychosis, cataract/glaucoma, infection (candidiasis, herpes, urinary tract infection) and pleural effusion. Such adverse events also reported somewhere else [36, 55] with different magnitude This adverse event variation could be due to difference in sample size and race of study participants, choice of immunosuppression and follow up period.

In the present study 40(35.1%) patients achieved complete remission, 51(44.7%) patients attained partial remission and 23(20.2%) patients had no remission to treatment at the end of the study. In addition, 7(6.14%) patients progressed/reached to ESRD and death occurred in 4(3.51%) patients. This is comparable to the findings of South Africa [50], India [37], Morocco [35], Senegal [41, 48] and Eastern India [51]. However, there are slight variations due to different study design, sample size, race of study participants, type of regimen, outcome criteria and diagnostic tests used.

Exacerbation or worsening of edema and relapse were found the common hospitalization events and reason for admission in LN patients during the study period in this study setting but the cause of death was not reported. A study by Ameh et al., reports that increased disease activity, kidney failure and infections were the common causes of mortality in LN patients [29]. This is also supported by other similar studies [35, 41].

There are different factors affecting the treatment outcome of LN patients depending on their race. In this study, AKI at onset, high SCr at six-months, no response at six-months to achieve complete remission and presence of flare were found the independent risk factors of poor treatment outcomes. The findings of this study comparable with other study [37]. However, a study by Momtaz et al., in Egypt reported that high baseline SCr, failure to achieve remission, hypertension, and nephritic flare were found the main risk factors for poor renal outcome [56]. Studies done in South Africa indicates that the factors associated with poor renal outcome in LN patients were elevated blood pressure, lack of complete remission at 6 months, nephrotic range proteinuria, low complement levels (C3 & C4) and positive double-stranded DNA [36, 43, 57]. Moreover, hypertension and nephrotic syndrome were factors of poor renal prognosis in many studies [40, 41, 51]. There may be slightly variations due to differences in the composite end points used such as drug choice, availability of diagnostic tests for histological identification, referral and follow up practice, racial variation which basically affects the treatment response and associated complications.

In this finding indicates that patients who develop AKI (*p* = 0.026) were found to be 4.8 times higher odds of poor prognosis than those without. This is similar to the finding of Senegal and South Africa reports [41, 43].LN patients who did not attain complete remission at six-months (*p* = 0.041) have 5% higher risk of poor renal prognosis. This is supported by a study in South Africa [36, 57] in which failure to achieve remission following induction therapy or lack of complete remission at 6 months results in poor renal prognosis.

Any history of relapse or flares (*p* = 0.004) during treatment of LN patients results in poor prognosis and to this effect needs additional immunosuppressive treatment. This is supported by a study done by Kammoun et al., and Sircar et al., in which high activity index score of LN was associated with poor renal prognosis [40, 51]. A study in Texas a high chronicity index is associated with poor response and MMF as a maintenance agent may improve the response to treatment [24]. But the use of MMF as maintenance therapy does not result any significant association in the present study. This may be affected by the sample size and availability of the medication.

### Limitation of the study

This study used a retrospective chart review of patients and many patients were excluded due to incomplete records and lost to follows up. The sample size used was small due to a single-center study as the kidney biopsy is limited to a few centers. In addition, a lot of patients did not afford repeated biopsy tests and decided their outcome by another alternative clinical diagnosis. All adverse drug events in this study were reported by physicians. However, any causality assessment was not done for these adverse drug event caused by either the LN medications or not. No evaluation was done for the non-pharmacologic intervention since the retrospective study and also difficult to find other important socio-demographic variables. Many patients shifted from one regimen to another due to interrupted supply and high cost of immunosuppressive medications and this may affect the treatment outcome.

## Conclusion

The treatment outcome of LN patients in this study was found comparable to other study findings, but relapse, treatment-related complications and adverse events of steroids and other immunosuppressive regimens needs close monitoring. AKI at onset, increased SCr at six-months, no response at six-months to achieve complete remission and presence of flares were predictors of poor treatment outcome.

### Recommendation

Prospective, multicenter, long-term study with a large sample size should be done so that the basic clinical and laboratory measurements are accurate and treatment-related adverse events will be easily identified. Health institutions and policymakers should work on the surveillance/early identification of SLE patients for renal involvement. Kidney biopsy should be done for all LN patients for specific management and minimize under diagnoses and under-reporting. Drug policymakers should work on the continuous availability of effective drugs for LN patients and help on the cost of the drug-using reimbursement policies or health insurance/ in accordance with the patients’ socio-economic status. Since LN is more prevalent in young women of childbearing age counseling with regard to contraception and pregnancy should be done early. Clinicians should adopt at diagnosing SLE and LN with proper referral and management system. Clinical pharmacists should work closely with nephrologists to minimize the drug-related adverse events so that maximize the treatment outcome.

## Supplementary Information

**Additional file 1.** 

## Data Availability

All relevant data are included in the article and uploaded as supporting information files. Extra data are accessed upon reasonable request of the corresponding author.

## Abbreviations

AKI: Acute kidney injury
ANA: Antinuclear Antibodies
Anti-dsDNA: Anti-double strand DNA
AZA: Azathioprine
CNIs: Calcineurin Inhibitors
CYC: Cyclophosphamide
CKD: Chronic Kidney Disease
CKD-EPI: Chronic Kidney Disease Epidemiology Collaboration
ESRD: End-Stage Renal Disease
GFR: Glomerular Filtration Rate
GN: Glomerulonephritis
FSGS: Focal Segmental Glomerulosclerosis
ISN/RPS: International Society of Nephrology /Renal Pathology Society
KDIGO: Kidney Disease Improving Global Outcomes
LA: Lupus anticoagulants
LN: Lupus Nephritis
NIH: National Institute of Health
MMF: Mycophenolate Mofetil
SLE: Systemic Lupus Erythematosus
SLICC: Systemic Lupus International Collaborating Clinics
SPHMMC: St. Paul’s Hospital Millennium Medical College
UPCR: Urine Protein to Creatinine Ratio
