# Supplementary material for: Management practice and treatment outcomes of adult patients with Lupus Nephritis at the Renal Clinic of St. Paul’s Hospital Millennium Medical College, Addis Ababa, Ethiopia
Source: BMC Nephrol. 2022 Jun 17;23:214. doi: 10.1186/s12882-022-02846-z (PMC9206350; doi:10.1186/s12882-022-02846-z)
Supplement: Supplementary file 1 — Additional file 1. [file 12882_2022_2846_MOESM1_ESM.pdf]

Annex

Data abstraction format

Part I: Socio-demographic characteristics

Patient MRN: ______________

1. Sex ☐Male ☐Female
2. If female, ☐Pregnant ☐Non-pregnant ☐Breast feeding
3. Age at LN diagnosis (year): ______________
4. Place of residence ☐Rural ☐Urban
5. Smoking status ☐ Smoker ☐Non-smoker ☐Not documented

Part II: Baseline patient clinical presentation

1. Initial confirmation/date of diagnosis of LN (SLE with renal involvement) _______________ date/month/year
2. Clinical presentation
   - ☐High blood pressure
   - ☐Periorbital puffiness
   - ☐Edema, Specify: ____________________________
   - ☐Other non-specific symptoms: ________________________________
3. Weight (kg) at diagnosis: _____________
4. Blood pressure (BP) at baseline: _____________mmHg, and at end of the study: ______________mmHg
5. Lupus class at first biopsy: ☐I ☐II ☐III ☐IV ☐V ☐VI
6. Repeated biopsy class: _________________

<u>Part III</u>: Medical history and LN complications

1. Presence of medical history Yes☐ No☐
2. Presence of LN complications Yes☐ No☐

If the response for the above question is yes, which of the following medical history or LN complications? (Can tick more than once)

**Comorbid conditions and LN complication**	Yes	No
**Any intrinsic renal disease**		
AKI (acute tubular necrosis, interstitial nephritis)		
Nephrotic syndrome		
Others(specify):		
**Any non-renal comorbidities**		
Antiphospholipid syndrome (APS)		
Lupus cerebritis		
Discoid lupus erythematous		
Drug induced lupus		
Infection-related glomerulonephritis		
Others(specify):		
**Complications**		
CKD/ESRD		
Others (thrombotic microangiopathy (TMA), cardiovascular complications, malignanancies)		

<u>Part IV</u>: Baseline and end of study pertinent laboratory investigations

1. Complete blood count (CBC)

Parameters	WBC	RBC	HGB	HCT	PLT	ESR
Baseline						
End of study						

2. Renal function tests (RFT)

Parameters	Serum creatinine (SCr)	Urea (BUN)	Estimated glomerular filtration (eGFR)
Baseline			
6th month			
End of study			

3. Urine analysis

Parameters	24-hr Urine Protein		Urine Protein	Urine WBC	Urine RBC	Urine bacteria	Urinary casts	Serum albumin	UPCR
	Non pregnant	Pregnant							
Baseline									
6th month									
End of study									

4. Serum electrolytes

Parameters	Sodium(Na)	Potassium(K)	Chloride(CL)	Calcium total	Phosphorus(Phos)
Baseline					
End of study					

5. Immunological investigations

Parameters	ANA	Anti-dsDNA	C3 Level	C4 Level	ANCA	RF
Baseline						
End of study						

6. Lupus anticoagulants (Lupus inhibitors)

Parameters	Lupus anticoagulants(LA)				
	Beta 2 Glycoprotein1-IgG	Beta 2 Glycoprotein1-IgM	LA1 screen	LA2 confirm	Ratio (LA1/LA2)
Baseline					
End of study					

7. Other antibodies tests

Parameters	Cardiolipin antibodies, IgG, IgM	Phospholipid antibody (APA), IgG, IgM	Beta 2 Glycoprotein1, IgA
Baseline			
End of study			

8. Coagulation profiles

Parameters	PT	INR	PTT
Baseline			
End of study			

9. Lipid profiles

Parameters	Total cholesterol	TG	HDL	LDL
Baseline				
End of study				

10. Liver function tests

Parameters	ALT	AST	ALP
Baseline			
End of study			

11. Glucose levels

Parameters	RBS	FBS	HgA1C
Baseline			
End of study			

Part V: Hospitalization events

1. Hospitalization events due to LN. ☐Yes ☐No
2. Reason for admission; ☐Due to LN flare/relapse ☐Due to other comorbidities
3. Presence/development of infection ☐Yes ☐No,

    3.1 If yes for no. 3 what antibiotics are given: ___________________________

<u>Part VI</u>: Medication management practice of LN complications and comorbidities (LN follow up management)

1. List of prescribed medications

**Type of medications**		**Dose and frequency**	**Start date**	**Stop date**	Yes	No
**Induction therapy/phase**						
Prednisolone						
Methylpredinsolone						
Cyclophosphamide						
Mycophenolate mofetil (MMF)						
Azathioprine						
Tacrolimus						
Rituximab						
**Maintenance therapy/phase**						
Prednisolone						
Mycophenolate mofetil (MMF)						
Azathioprine						
Tacrolimus						
**Kidney protective regimens**						
ACEIs						
ARBs						
**Other medications (adjunctive treatments)**						
Chloroquine or Hydroxychloroquine						
Others (specify if pertinent to LN):						

2. Total duration of treatment for LN up to end of data collection in months: ____________________

Part VII: If any medications are adjusted/stopped/changed during the study period,

Medication name	Reason(ADR, relapse/flare, pregnancy, availability/cost issue)	Description of the ADR if reported

<u>Part VIII</u>: Outcome measures/end points

1. Complete renal remission (CR)
    1.1. Decline or reduction in proteinuria to < 0.5g/g measured as UPCR from a 24-hr urine collection: Yes □ No □
    1.2. Stabilization or improvement in kidney function (±10-15% of baseline): Yes □ No □
2. Partial renal remission (PR)
    2.1. Reduction in proteinuria by at least 50% and to <3g/g measured as UPCR from a 24-hr urine collection : Yes □ No □
    2.2. Stabilization or improvement in kidney function (±10-15% of baseline): Yes □ No □
3. No response/non responders (NR)
    3.1. Failure to achieve a complete or partial remission: Yes □ No □
        3.1.1. Worsening of kidney function: Yes □ No □
        3.1.2. Re-biopsy (activity present or absent): Yes □ No □
4. Worse outcomes
    4.1. Progression to ESRD: Yes □ No □
    4.2. All-cause mortality: Yes □ No □
